# The behaviour of overweight dogs shows similarity with personality traits of overweight humans

**DOI:** 10.1098/rsos.172398

**Published:** 2018-06-06

**Authors:** Ákos Pogány, Orsolya Torda, Lieta Marinelli, Rita Lenkei, Vanda Junó, Péter Pongrácz

**Affiliations:** 1 Department of Ethology, Biological Institute, Eötvös Loránd University, Budapest, Hungary; 2 Department of Comparative Biomedicine and Food Science, University of Padova, Padova, Italy

**Keywords:** overweight, dog, personality, cognitive bias, food reward

## Abstract

Excessive food intake and the resulting excess weight gain is a growing problem in human and canine populations. Dogs, due to their shared living environment with humans, may provide a beneficial model to study the causes and consequences of obesity. Here, we make use of two well-established research paradigms (two-way choice paradigm and cognitive bias test), previously applied with dogs, to investigate the role of obesity and obesity-prone breeds for food responsiveness. We found no evidence of breed differences in food responsiveness due to one breed being more prone to obesity than another. Breed differences found in this study, however, can be explained by working dog status, i.e. whether the dog works in cooperation with, or independently from, humans. Our results also confirm that overweight dogs, as opposed to normal weight dogs, tried to maximize food intake from the higher quality food and hesitated to do the task when the food reward was uncertain. These results are very similar to those expected from the parallel models that exist between certain personality traits and being overweight in humans, suggesting that dogs are indeed a promising model for experimentally investigating obesity in humans.

## 1. Background

Just as in humans, obesity and overeating represent a steadily growing problem in companion dogs. A recent survey from the USA reported 34% of the examined dogs being overweight or obese [1]. Three major factors which contribute to the excess fat accumulation in dogs are: genetic predisposition, reproductive management (i.e. neutering of female dogs) and suboptimal dietary/exercise conditions [2].

Among researchers, there is a vivid interest in the possible causes leading to obesity in dogs. First, dogs cohabit with humans; therefore, members of the two species arguably experience approximately the same external factors that cause obesity, which in turn gives less opportunity for differences caused by gene–environment interactions. Because of this, dogs may serve as a more suitable model species than rodents for investigating the causes and consequences of obesity in humans [3,4,5]. Second, the negative effects of obesity on dogs' health and welfare represent an important problem on its own. In contrast to human studies (e.g. [6,7]), we mostly lack information on the behavioural/motivational causes of overeating and obesity in dogs (but see [8]), while there is a growing body of literature about the complex relationship between human obesity and certain psychiatric/mood disorders, including depression (e.g. [9]). It was found that subjects with binge eating problems show characteristic symptoms of weak inhibitory control and attention focus [10]. More importantly, several studies have shown that obesity coincides with particular forms of cognitive biases, such as interest towards unhealthy food items [11]. Cognitive biases may be tested by various methods (e.g. ‘emotional Stroop test'; ‘ambiguous stimulus test'), where the core hypothesis is that an individual's background affective state biases its decision making in a task that is not directly related to the aforementioned inner state (e.g. [12]).

While genetic predisposition in dogs is considered an inevitable factor behind excess weight [13], other factors including food-related behaviours, incentive value of food and the motivation to eat have received very little attention in terms of canine obesity. In humans, the cognitive approach towards obesity emphasizes the social implications of food as reward and focuses on the behavioural responses to food rewards [14].

Although there is a vivid debate over the possible benefits and negative consequences of extrinsic rewards on human creativity and motivation [15,16], the so-called primary rewards, such as food, drink and positive social interactions, are considered almost unequivocally necessary for achieving higher motivation level and learning performance in animals. Testing the effect of reward quality on dogs proved to be surprisingly difficult, according to the last few decades' ethological research. Although dogs are definitely motivated in participating in tasks where food or object (e.g. toy) rewards are involved, their performance is often more strongly affected by social factors such as human communicative actions than the quality, quantity or the presence of a reward; therefore, it seems reasonable to conclude that food may not be the only motivation during different kinds of problem-solving tasks.

Food responsiveness (defined as part of someone's appetite in terms of willingness to eat [17]), voracity and indiscriminate food intake are commonly considered attributes of particular dog breeds, which in turn provide a higher proportion of obese dogs than other, non-obesity-prone breeds. However, there is a surprising lack of empirical studies that would investigate this assumption. In a controlled feeding experiment, Hewson-Hughes *et al*. [18] tested whether dogs from five breeds (from toy to giant size) chose differently, when they had the opportunity to ‘compose' the macronutrient ratio of their meals. Interestingly, all breeds showed a strikingly similar preference for a particular protein–fat–carbohydrate ratio, although each breed ate quantitatively more than the actual nutritional requirement. Recently however, Raffan *et al*. [19] showed in a questionnaire study, that dog owners experienced marked differences among dog breed groups in the level of food responsiveness. According to this survey, hounds and gundogs showed the highest food responsiveness, while pastoral and working dog breeds were the least motivated to eat. This result is in line with studies investigating breed differences in obesity (e.g. [20,21]). The hound and gun dog group (such as Labrador retrievers and Basset hounds) were those that were most prone to obesity; meanwhile, working breeds (such as the Doberman) were usually normal weight. Labrador retrievers were also the first dog breed in which a gene mutation was found recently [13], affecting not only the accumulation of adipose tissue, but also the food responsiveness of obese dogs (the latter result was derived via a questionnaire survey). Whether this (or a similar) gene mutation can be found across a wide range of breeds is currently unknown [22], but it provides a potential proximate mechanism for any breed differences in obesity.

In this study, we tested the behaviour of overweight and normal weight dogs from obesity-prone and not obesity-prone breeds in two experiments in which food motivation plays an important role. In the first experiment, we used the so-called two-way choice paradigm (e.g. [23–25]), in which the experimenter indicates the location of hidden food reward with a pointing gesture. While at the indicated location dogs can always find food reward with a low incentive value, depending on the experimental group, the non-indicated (alternative) location contained either nothing or a high-quality reward. Dogs did not know the content of the bowls; it was only revealed to them after they made a choice. Our prediction was that overweight and obesity-prone dogs would perform more willingly in this test paradigm, even for lower quality food. A complementary hypothesis, however, suggests a contrasting effect, in that the higher food responsiveness of these dogs would result in a weaker point-following response especially in that group where the non-indicated bowl contained a higher quality food.

In the second experiment, we used one of the so-called cognitive bias paradigms (e.g. [26,27]). This method requires that subjects are first trained to expect at one site (left or right, consistently) the container always being rewarded, while expecting it to be empty at the opposite site. Then, in the test phase, the bowl is placed halfway between these two sites (i.e. at an ambiguous position). Based on the differences in obesity proneness among dog breeds [13,20,21], we expected that overweight and obesity-prone dogs would show higher/non-selective food responsiveness, so they will approach the ambiguous location faster than the normal weight and non-obesity-prone dogs (i.e. they will show ‘positive expectancy'). However, we can formulate an alternative hypothesis based on similarities in ‘pessimistic attitudes' between human subjects with higher body mass indices [28] and obese dogs. This would predict contrasting behaviour, i.e. overweight canine subjects would rather show a negative cognitive bias.

## 2. Material and methods

### 2.1. Subjects and assessment of body condition

Subjects were healthy adult family dogs from the following dog breeds: ‘obesity prone' breeds—Beagle *N* = 21, Golden Retriever *N* = 8, Labrador Retriever *N* = 14; ‘not obesity prone breeds'—Border Collie *N* = 24, Mudi *N* = 24. Based on the similarity of breed histories, working functions and their proneness to obesity (e.g. [29]), for the subsequent statistical analysis, we merged the Labrador and Golden Retrievers to a single ‘Retriever' group. All of the dogs that participated in the experiments were older than one year, and accepted food in novel situations (a pre-requisite in our test). The body condition of the subjects was determined by a combination of visual inspection and palpation (assessments were performed by one of the authors with a DVM degree, O.T.). We used a three-level scoring system (thin, normal, overweight); however, in our sample there were no thin dogs. Body condition score 2 (BCS 2) dogs were normal weight: the ribs of the animals were easy to touch, they were covered by a thin layer of fat under the skin and the waist section was also visible. BCS 3 dogs were overweight or obese: it was difficult or impossible to locate the ribs because of the thick layer of fat under the skin and the waist section was almost undetectable. In our sample, the following dogs were found to be overweight: Retrievers *N* = 8 (38%); Mudis *N* = 2 (8%); Border Collies *N* = 0 (0%); Beagles *N* = 6 (29%). With the exception of 12 dogs (which we could not invite back to the second test), each subject was tested in the two-way object choice and cognitive bias tests.

### 2.2. Experimental procedure

**Figure 1.**
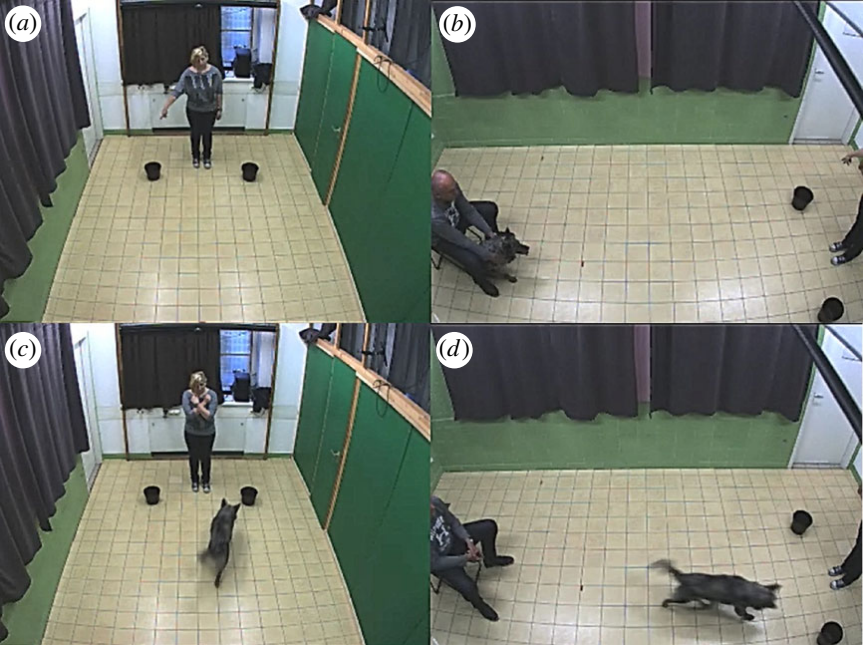
Arrangement of the two-way object choice test. (*a*) Experimenter points at the bowl that contains the low-quality reward; (*b*) owner keeps the dog at the start while the experimenter is pointing. (*c*,*d*) Experimenter finished pointing and the dog was released by the owner. The dog approaches the non-indicated bowl in this case.

The subjects were tested indoors, in an empty experimental room, where only the dog, the dog's owner (O) and the experimenter (E) were present during the tests (figure 1). The tests were videotaped, and the recordings were behaviourally coded using the Solomon coder (http://solomoncoder.com, by András Péter) [30] and Observer XT (Noldus Information Technology).

Each dog participated in the two tests in a fixed order: first in the two-way object choice test and then in the cognitive bias test. Testing in fixed order was necessary because the cognitive bias test may form side bias in the subjects which would confound a subsequent two-way object choice test. Before the two tests began, each dog participated in a pre-test (‘food preference' henceforth), to ensure that without any manipulation, dogs preferred high incentive value food over low incentive value food used in the subsequent tests.

### 2.3. Pre-test for food preference

After entering the testing room, the subject had 1–2 min off leash to explore the experimental room. When the time expired, two types of food were offered to the dog in a non-transparent plastic plate. One of them was a low incentive value food (carrot or orange) and the other was a high incentive value food (Frolic dog reward treat). The assigned locations of the different quality foods were marked by a piece of tape, approximately 20 cm from each other. The relative position of the food items (i.e. left or right) was randomly changed by turning the plate by 180°. The two food items were presented simultaneously. The E offered the plate with the pieces of food to the dog holding the plate in her hand. The dog had to choose ten times. The type of food the dog ate seven or more times was considered as ‘preferred', and the other type was considered as ‘non-preferred'. Between tests with different dogs, the plate was cleaned with water and dish soap.

### 2.4. Two-way object choice test

#### 2.4.1. Pre-test phase

The O held the dog by its collar/harness at the start point. The E placed one brown, non-transparent plastic bowl (round, plastic flower pot, 20 cm tall and 20 cm wide) on the floor approximately 1 m from the dog. The E conspicuously dropped a piece of the preferred food into the bowl. After this, the O let the dog free and if it was needed, verbally encouraged it to eat the food. This action was repeated once more.

#### 2.4.2. Test phase

Dogs were randomly allocated to two experimental groups: an empty alternative group (EA) and a reward alternative group (RA), based on the content of the non-indicated bowl. At the beginning of each trial, the dog was held by its owner at the start point. Invisibly, for the dog and for the O, the E put a piece of low incentive value food in one of the two identical bowls; and this was the indicated bowl in the experiment, so that it always contained a low incentive value food regardless of the experimental group of the dog. In the other (alternative) bowl, either a high incentive value food was placed for each trial of a given dog (RA group) or no food was placed (EA group). The high incentive value food in the RA group was placed into the alternative bowl the same way as the low incentive value food was placed into the indicated bowl (i.e. invisibly for the dog and the O). The bowls were marked on their inside wall so that the same bowl was used for the same type of food consistently. After placing the reward(s) in the bowls, the E turned towards the dog and the O and held both bowls in front of her body then exchanged the two bowls between her hands a few times. Then the E put the two bowls simultaneously on the floor on her left and right side, 1.5 m from each other and 2.6 m from the subject. The left and right position for the indicated and the alternative bowl was varied in a semi-random order (the same bowl was placed no more than two times on the same side, and during the first two trials in each session the indicated bowl's position was alternated). Then the E stood up and held her hands in front of her chest and called the dog by its name to attract the dog's attention. After the E established eye contact with the subject, she pointed towards the bowl with low incentive value food (‘indicated' or ‘correct' location) for 2 s with extended arm (figure 1). After the pointing cue, the E put her hand back to the front of her chest and the dog was released by the O (momentary distal pointing [25]). If the subject did not leave the start position for 3 s after it was released, the E repeated the pointing gesture once more. As soon as the dog reached one of the bowls, the E quickly took away the other one to prevent the dog from examining both bowls. The dog was allowed to eat the content of the chosen bowl. The O called the dog back to the start point and the next trial started. The maximum trial number in this experiment was 20. The dogs had to choose within 30 s. If a dog did not choose within this time period in two subsequent trials, this experiment was terminated for the given dog, and the last two trials (in which the dog did not choose) were excluded from statistical analyses. We had two alternative predictions: (1) obese dogs/obesity-prone breeds will show higher success rates in choosing the indicated bowl (they maximize the reward intake even if it is a low quality one); or (2) obese dogs/obesity-prone breeds give up easier on the indicated bowl (low quality food), especially after they noted that the alternative bowl contains high-quality food.

### 2.5. Cognitive bias test

Following a minimum one-week interval after the two-way object choice test, the same subjects were invited to participate in the cognitive bias test.

#### 2.5.1. Training phase (location discrimination task)

At the beginning of each trial, the O held the dog by its collar/harness on the start point. In the case of each subject, one side of the experimental room was allocated to the ‘rewarded location' and the other side was the ‘non-rewarded location'. These were kept constant throughout the entire procedure for the given subject. Left and right side was designated as ‘rewarded' or ‘non-rewarded' location in an equal proportion of the subjects. The bowl at the ‘rewarded location' always contained a piece of the preferred food, while at the non-rewarded location the bowl was always empty. Before each trial, the E placed one bowl on the ground either to the ‘rewarded' or to the ‘non-rewarded' location in semi-random order. The bowl was 3 m away from the subject and neither the dog nor the O was able to see the content of the bowl from the start point. After placing the bowl, the E went behind the O and touched his/her shoulder, signalling that the dog can be released. The E measured the latency between the releasing of the dog to reaching the bowl using a digital stopwatch. The trial was finished when the dog reached the bowl, or 30 s after the O released the dog (whichever happened first). The training phase ended when the threshold criterion was reached by the subject: the latencies of the last five ‘rewarded' trials were shorter than in the last five non-rewarded trials.

#### 2.5.2. Test phase

Test trials started immediately after the training phase. Three trials were conducted in a fixed order; after a rewarded and non-rewarded trial (in random order), the bowl was placed halfway between the rewarded and non-rewarded locations (ambiguous location). Half of the dogs were tested in rewarded–non-rewarded–ambiguous trial order and the other half in non-rewarded–rewarded–ambiguous trial order. The testing procedure was otherwise the same as described above in the training phase. Our two, competing predictions were: (1) obese dogs/obesity-prone breeds will show positive expectancy in the case of the ambiguous location, because they have higher food responsiveness; or (2) these dogs would be rather reluctant in approaching the ambiguous location due to their pessimistic predisposition.

### 2.6. Statistical analyses

Statistical analyses were carried out in R statistical environment (https://www.R-project.org, v. 3.2.3, R Core Team). We used linear mixed-effects models (LMMs; R package ‘lme4'), binomial generalized linear models (GLMs), binomial generalized linear mixed models (GLMMs; R package ‘lme4') and mixed-effects Cox models (MECMs; R package ‘come').

In all analyses, backward model selection was based on Akaike information criterion (AIC), so that the model with the lowest AIC value was kept and we considered a model better whenever delta AIC was ≥2. The effects of explanatory variables were analysed by *F*-tests (in LMMs) and likelihood ratio tests (in GLMs, GLMMs and MECMs); we provide *F* or *χ*^2^ and the corresponding *p*-values of tests of models with and without the explanatory variable. For non-significant explanatory variables that were in the focus of our research, we provide test statistics before exclusion from the final model. Parameter estimates (for GLMs and GLMMs) and hazard ratios (Exp[*β*], for MECMs) with 95% confidence interval are also provided between levels of a given significant fixed effect.

#### 2.6.1. Two-way object choice test

We focused on three behavioural responses in the two-way object choice test, separately: endurance, latency to choosing, and preference for the indicated bowl. Endurance (binary response: whether or not the dog completed the 20 trials) was analysed using binomial GLMs. The full model included breed (factor with four levels: Beagle, Retriever, Border Collie, Mudi), body condition (factor with two levels: overweight, normal), experimental group (i.e. non-indicated bowl content; factor with two levels: empty, rewarded) and all possible two-way interactions between these fixed effects.

Latency to choosing (time spent between the moment that the owner released the dog and when it reached any of the two offered bowls) was inverse-transformed for normalizing residuals, and then analysed in LMMs. The full model included the same independent variables as the endurance analysis (breed, body condition, experimental group) in addition to trial as a covariate and dog ID as a random term. Initial models also included all possible two-way interactions between independent variables. A small number of trials (36 of 1593 or 2.3%) in which dogs did not go to any of the bowls within 30 s of the trial were treated as censored observations and were excluded from this analysis.

Preference for the indicated bowl (i.e. number of times choosing it) was analysed using binomial GLMMs with choosing and not choosing the indicated bowl as success and failure, respectively. The full model included the same fixed effects as the latency to choosing analysis (i.e. breed, body condition, experimental group, trial and their two-way interactions) and dog ID as a random term.

#### 2.6.2. Cognitive bias test

In the cognitive bias test, probability of reaching the bowl was analysed in MECMs with latency to reaching the bowl as response and reaching as terminal event. Dogs that did not reach the bowl within 30 s of the trial were treated as censored observations. Full models included test trial (factor with three levels: rewarded, non-rewarded and ambiguous) and body condition (factor with two levels: normal, overweight) and breed (factor with four levels: Beagle, Retriever, Border Collie, Mudi) and all possible two-way interactions between them as fixed effects, in addition to dog ID as a random effect.

#### 2.6.3. Inter-coder reliability

To assess the reliability of behavioural coding, the video footages of 12 randomly selected dogs that participated in both experiments were independently coded by a second observer. Inter-coder reliability was then investigated using the R package ‘rptR' [31]. This analysis revealed strong agreement between the two observers; in the two-way object choice test, endurance and preference (see above) were scored identically by the two observers (i.e. 100% agreement between them), and the latency was scored almost identically as well (repeatability estimate with 95% CI: *r* = 0.996 [0.995; 0.997], *p* < 0.001). Similarly, latencies coded by the two observers in the cognitive bias test were highly repeatable (*r* = 0.999 [0.997; 0.999], *p* < 0.001).

## 3. Results

### 3.1. Two-way object choice test

**Figure 2.**
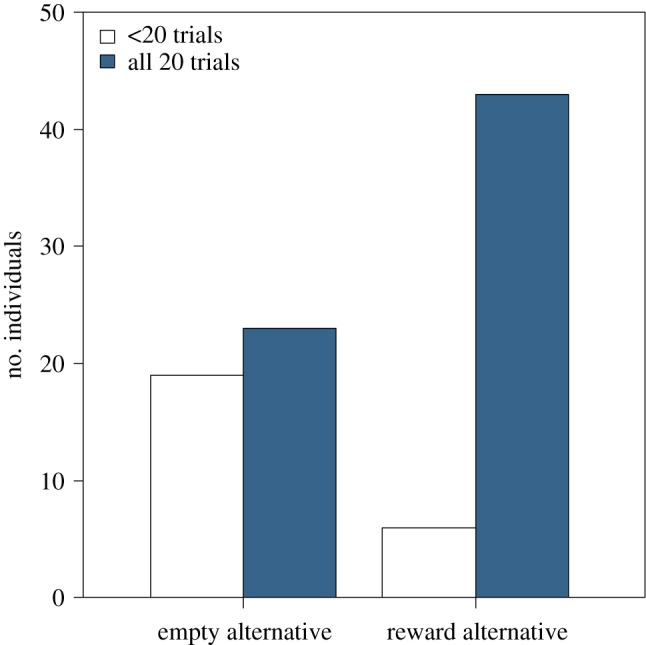
Endurance, investigated as whether or not the dog completed all 20 test trials. Results from the two-way object choice test, based on experimental groups (i.e. whether or not the non-indicated alternative bowl contained a preferred food item). Significantly more dogs finished all 20 trials when the non-indicated bowl contained high-quality food. *N *= 91; binomial GLM of endurance, effect of experimental group: *p *< 0.001.

From the 91 dogs involved in the study, 66 (or 73.33%) completed all 20 trials (range: 3–20, mean ± s.d. = 18.0 ± 4.2 trials per dog). The endurance analysis revealed experimental group differences (binomial GLM of endurance, effect of experimental group: *χ*^2^(1) = 12.72, *p* < 0.001; figure 2), because dogs that were tested in reward alternative test conditions were more likely to complete all 20 test trials than dogs that were tested in the empty alternative conditions (*b* = 1.78 [0.78; 2.90], *z* = 3.33, *p* < 0.001). Endurance was not influenced by body condition and was not different between breeds (both *p* > 0.134).

The analysis of latency to choosing revealed that the test trial had an experimental group and breed-specific effect on how fast dogs reached any of the two offered bowls, reflected in significant two-way interactions (LMM of latency to choose (inverse transformed), trial × experimental group interaction: *χ*^2^(1) = 100.84, *p* < 0.001; trial × breed interaction: *χ*^2^(3) = 24.16, *p* < 0.001). The experimental group-specific effect was driven by dogs choosing faster with increasing trial number in reward alternative, as opposed to empty alternative conditions (effect of trial, reward alternative versus empty alternative experimental groups: *b* = 0.010 [0.008; 0.012]). The breed-specific effects were mainly driven by the trial having different effects in Border Collies as opposed to the other three breeds (effect of trial, Mudi versus Retriever: *b* = 0.001 [−0.002; 0.004]; Border Collie versus Retriever: *b* = 0.006 [0.003; 0.008]; Beagle versus Retriever: *b* = 0.001 [−0.002; 0.003]). Body condition had no effect on latency to choosing (*p* > 0.206).

**Figure 3.**
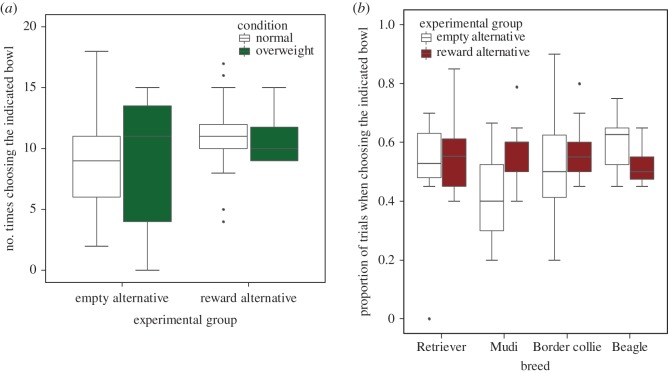
Number and proportion of test trials when dogs chose the indicated bowl in the two-way object choice test. Results are based on (*a*) experimental group and body condition interaction (binomial GLM, *p* = 0.047) and (*b*) experimental group and breed interaction (*p *= 0.016). Overweight dogs (*a*) and Beagles (*b*) chose less frequently the indicated bowl if the alternative bowl contained the high-quality reward. In the empty alternative test conditions, the non-indicated bowl contained no food item, whereas in the reward alternative test conditions, it contained a preferred food item (as opposed to the non-preferred, low-incentive value food present in both test conditions in the indicated bowl). *N* (breed of dog, in parentheses the number of overweight subjects)—Retriever = 22 (8); Mudi = 24 (2); Border collie = 24 (0); Beagle = 21 (6).

Experimental group had a body condition-specific and breed-specific effect on preference for the indicated bowl, i.e. the proportion of trials when choosing it (binomial GLM of number of times choosing the indicated bowl as opposed to number of times not choosing it, experimental group × body condition interaction: *χ*^2^(1) = 3.95, *p* = 0.047; experimental group × breed interaction: *χ*^2^(3) = 10.28, *p* = 0.016; figure 3*a*). The body condition-specific effect of experimental groups was driven by overweight dogs choosing less often the indicated bowl in the reward alternative test conditions than normal weight dogs (overweight versus normal body condition, empty alternative versus reward alternative: *b* = −0.61 [−1.22; −0.01], *z* = −1.98, *p* = 0.048; figure 3*b*). Breed-specific effects of experimental groups were mainly due to Beagles choosing the indicated bowl less frequently in the RA group than the other three breeds (reward alternative versus empty alternative, Retriever versus Mudi: *b* = 0.39 [−0.22; 1.00], *z* = 1.26, *p* = 0.209; Retriever versus Border Collie: *b* = −0.24 [−0.86; 0.37], *z* = −0.78, *p* = 0.435; Retriever versus Beagle: *b* = −0.48 [−1.07; 0.10], *z* = −1.62, *p* = 0.104).

### 3.2. Cognitive bias test

**Figure 4.**
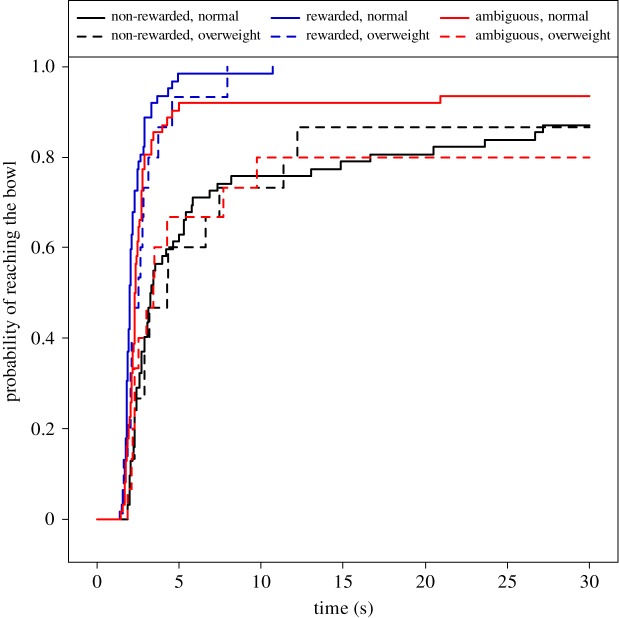
Probability of reaching the bowl in cognitive bias tests of normal and overweight dogs. The *x*-axis represents time spent until occurrence of reaching the bowl, whereas the *y*-axis represents the cumulative proportion of dogs that had already reached the bowl. Each dog was tested in three test trials, half of the dogs in non-rewarded–rewarded–ambiguous order and the other half in rewarded–non-rewarded–ambiguous order. Regardless of their body condition, dogs reached the rewarded bowl more likely than the non-rewarded one. Overweight dogs reached the ambiguous bowl with a higher probability than the normal weight dogs did. *N *= 91; mixed-effect Cox models, *p *= 0.026.

Body condition had a test trial-specific effect on the probability of reaching the bowl (MECM, effect of body condition × test trial interaction: *χ*^2^(2) = 7.31, *p* = 0.026; figure 4). This interaction was mainly driven by a decreased probability of reaching the bowl in overweight, as opposed to normal weight, dogs in the ambiguous test trials compared to the non-rewarded test trials (overweight versus normal body condition, non-rewarded versus ambiguous test: Exp[*β*] = 0.28 [0.11; 0.71], *z* = −2.67, *p* = 0.008). A similar, albeit smaller, difference between the effect of body condition in rewarded as opposed to non-rewarded tests also contributed to this interaction (overweight versus normal body condition, non-rewarded versus rewarded test: Exp[*β*] = 0.40 [0.17; 0.96], *z* = −2.05, *p* = 0.040).

**Figure 5.**
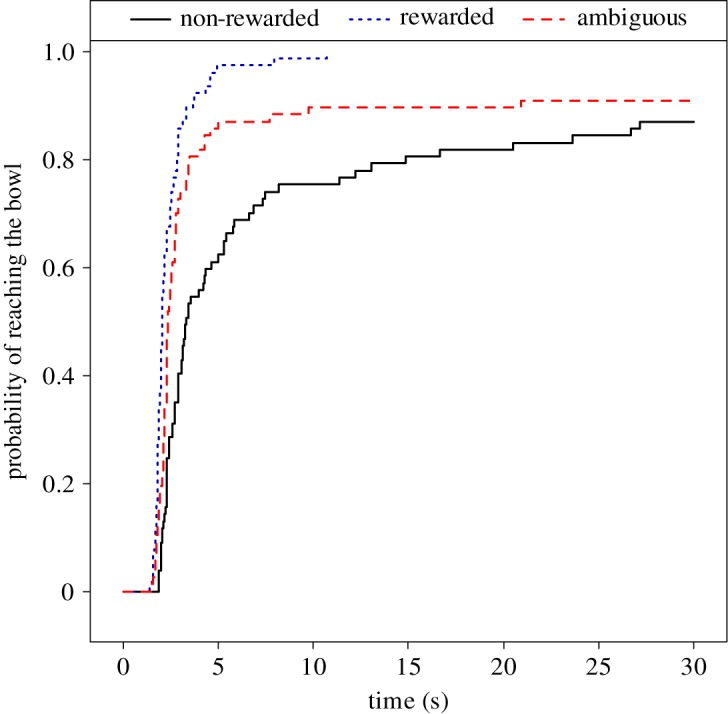
The effect of test trial (non-rewarded, rewarded or ambiguous) on probability of reaching the bowl in cognitive bias tests of dogs. The *x*-axis represents time spent until occurrence of reaching the bowl, whereas the *y*-axis represents the cumulative proportion of dogs that had already reached the bowl. Dogs reach the ambiguous bowl with a higher probability than the non-rewarded bowl, but with a lower probability than the rewarded bowl. *N *= 91, mixed effect Cox models, *p *< 0.001.

The probability of reaching the bowl was also different between test trials (MECM, effect of test trial: *χ*^2^(2) = 94.38, *p* < 0.001; figure 5). The difference was driven by increased probabilities of reaching the bowl in the rewarded and ambiguous, as opposed to the non-rewarded tests, with probability of the ambiguous tests being between those of the rewarded and non-rewarded tests (ambiguous versus non-rewarded tests: Exp[*β*] = 4.02 [2.57; 6.31], *z* = 6.07, *p* < 0.001; rewarded versus non-rewarded: Exp[*β*] = 10.74 [6.71; 17.19], *z* = 9.88, *p* < 0.001).

**Figure 6.**
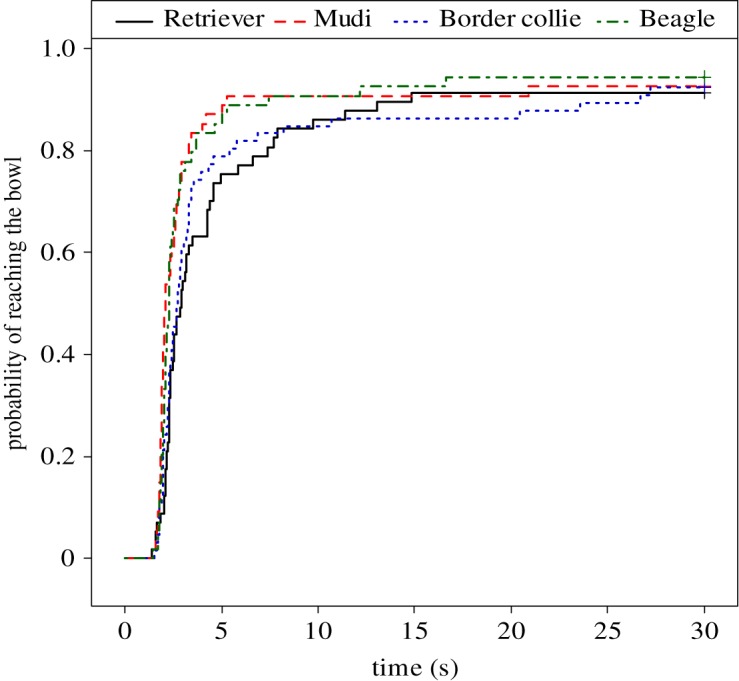
Probability of reaching the bowl in cognitive bias tests of four breeds of dogs. The *x*-axis represents time spent until occurrence of reaching the bowl, whereas the *y*-axis represents the cumulative proportion of dogs that had already reached the bowl. Mudis and Beagles reached the bowl with a higher probability than the Retrievers and Border Collies did. *N*: Retriever = 22; Mudi = 24; Border Collie = 24; Beagle = 21. Mixed-effect Cox models, *p *= 0.015.

In addition, this analysis revealed breed differences in the probability of reaching the bowl (*χ*^2^(3) = 10.52, *p* = 0.015; figure 6), because Mudis and Beagles reached the bowl with a higher probability than Retrievers and Border Collies (Mudi versus Retriever: Exp[*β*] = 2.89 [1.14; 7.35], *z* = 2.23, *p* = 0.026; Border Collie versus Retriever: Exp[*β*] = 1.19 [0.47; 3.02], *z* = 0.37, *p* = 0.710; Beagle versus Retriever: Exp[*β*] = 2.98 [1.26; 7.02], *z* = 2.49, *p* = 0.013).

## 4. Discussion

In two separate experiments, the possible effects of body condition and breed were tested on dogs' performance in tasks where food responsiveness can be important. In the two-way object choice test, we found that independent of their breed and body condition, dogs were less likely to quit the test (that is, they were more prone to complete all 20 trials) when the alternative pot contained (high-quality) food. Similarly, dogs in the rewarded alternative pot group chose faster towards the end of this test, compared to the EA group. Again, body condition did not affect the latencies to choose. However, body condition had an effect (in interaction with the reward-content of the non-indicated bowl) on how often dogs chose the indicated bowl. Overweight dogs less often followed the experimenter's pointing when the alternative bowl contained a reward. A similar effect was found in the case of dog breeds, because compared to the other breeds, Beagles were less likely to choose the indicated bowl when the non-indicated bowl contained food.

In the cognitive bias test, we found a marked effect of body condition, because overweight dogs reached the ambiguous bowl with a lower probability than the normal weight dogs did. In support of expectations from the test paradigm, dogs in general reached the bowl at the non-rewarded location with the lowest, and the bowl at the rewarded location with the highest probability, with the likelihood of reaching the bowl at the ambiguous location falling between these two. Breed differences in the probability of reaching the ambiguous bowl were also apparent in this experiment, as Beagles and Mudis were more likely to reach it than Border Collies and Retrievers.

In contrast to some of our *a priori* hypotheses, overweight dogs did not show unselective food responsiveness. It was recently found that obese people, even when satiated (i.e. ‘not hungry') showed increased interest in food images compared to normal weight controls [32]. Based on this finding, one could predict that overweight dogs would follow human pointing more frequently, even if the signalled bowl contained low-quality reward (i.e. ‘do not abandon the safe food' strategy); as well as a more positively biased response towards the ambiguous location in the cognitive bias test (i.e. ‘if it is different than negative, it has to be positive' strategy). However, in our tests, overweight dogs reacted more sensitively to the presence of a high-quality reward in the non-indicated bowl than the normal weight dogs did. In the cognitive bias test, overweight dogs were less eager to approach the ambiguous location when compared with the normal weight subjects. The behaviour of overweight dogs indicated that these subjects were more responsive to the possibility of consuming more rewarding food in the two-way object choice test; and they showed a more conservative/cautious (or ‘pessimistic') strategy in the cognitive bias test. Although the results of the two-object choice test could theoretically be explained by obese participants being less able to focus their attention, similar to humans [10], such an explanation would not be in line with the results of the cognitive bias test (where the approach pattern of overweight dogs showed that these subjects must have had a clear picture of the positively reinforced location).

The connection between being overweight and showing negative cognitive bias (pessimism) is often explained on the basis of the relationship between depression and obesity in humans [33,34]. However, with regard to our canine subjects, a model considering the association between particular personality traits and obesity seems to be a more appropriate approach, in line with recent research discovering the existence of human-analogous personality structure in dogs (e.g. [35,36]). In human participants, personality traits were found to play an important role both in terms of risks and in terms of protective factors in the development of overweight problems. Neuroticism, impulsivity and sensitivity to reward turned out to be risk factors, but conscientiousness and self-control have been shown to contribute as protective traits in relation to weight gain [37]. From our aspect, it is especially interesting that impulsivity (a personality trait also found in dogs [38,39]) shows strong connection to obesity and binge eating disorder. Mobs *et al*. [38] found that overweight and obese subjects had higher levels of urgency, lack of perseverance and sensitivity to reward, which are three from the five dimensions of impulsivity [40]. In our tests, overweight dogs also responded with higher sensitivity to the reward, because they were more often abandoning the low-quality food indicated by the experimenter for the sake of the high-quality food in the non-indicated bowl. This behaviour may also be connected to the above-mentioned lack of perseverance in obese subjects. If so, overweight dogs might have shown less perseverance in following human pointing cues (that lead to an unsatisfying reward); therefore, they might have been able to detect the presence of an alternative reward earlier in the non-indicated bowl. Negative cognitive bias can also be explained on the basis of higher sensitivity to reward in the overweight dogs, because the ambiguous location could have been assessed as a ‘less promising' food source compared to the ‘positive' location.

We found moderate effect of dog breeds in both experiments. Beagles behaved similarly to the overweight dogs in the two-way object choice test, by less often choosing the indicated bowl when the non-indicated bowl contained reward. Beagles are obesity-prone dogs (e.g. [1]); therefore, their present strategy (i.e. maximizing energy intake by increased responsivity to higher quality food) could contribute to the overweight problems in this breed. However, we should also consider that the Retrievers in our sample did not show a similar reaction as the Beagles did, although in the Retriever group we had a high occurrence of overweight subjects. Variable responses of the two obesity-prone breeds could be explained by differences in their original function. While Beagles were selected for hunting on their own (i.e. chasing game without human assistance), Retrievers traditionally work in close interaction with humans. It was found that in a two-way choice task, similar to the one used in our experiment, cooperative breeds follow human pointing with higher fidelity than the independent working breeds do [41].

The prediction that obesity-prone breeds would react similarly in the cognitive bias experiment was not supported. Retrievers and Beagles responded differently, and as was shown earlier, overweight subjects in general showed negative bias in the ambiguous test condition. If we examine the results from the aspect of selection for independent or cooperative work, we can conclude that both breeds with lower probability to reach the ambiguous location (Border Collies and Retrievers) belong to the cooperative breeds [41], meanwhile cooperative (Mudi—a Hungarian herding dog breed) and independent (Beagle) dog breeds reached the ambiguous location with higher probability. This suggests that cooperative breeds may be characterized mostly by a negative cognitive bias, resulting from the stronger effect of being reinforced at the positive location. Independent working breeds, however, may approach the ambiguous location with a higher probability, because their behaviour is less bound to patterns reinforced by humans; this behavioural pattern and explanation are in line with the results of our two-way object choice test.

## 5. Conclusion

The results of our experiments showed that normal and overweight dogs behave differently in tasks that involve food and interaction with humans as motivation. We found that overweight dogs either try to maximize the intake of higher quality food (two-way object choice test), or they hesitate when they face a formerly untried location for obtaining food (cognitive bias test). Food choice patterns in humans with overweight/obesity problems are complex and they are influenced by various genetic and environmental factors, including social learning and cultural transmission [42]. However, it is a general phenomenon that overweight/obese subjects show attraction towards energy-dense foods [43]. In our study, dogs proved to be a useful model species to test characteristic patterns of food responsiveness in normal and overweight subjects, showing similar strategies to those expected from human subjects. Being an obesity-prone breed, however, did not have an effect, because, we argue, breed differences reported in our study are rather driven by the cooperative/independent working dog status of these particular breeds. Furthermore, we found that an independent breed (the Beagle) showed similar food choice strategy to overweight dogs in general, adding a potential new risk factor to the already created long list for canine obesity (e.g. [44]).

## Supplementary Material

Raw research data 1

## Supplementary Material

Raw research data 2

## Data accessibility

All data are available in the electronic supplementary material.
